# Foreign Correspondence

**Published:** 1889-09

**Authors:** L. C. Bryan

**Affiliations:** Basel, Switzerland


### Foreign Correspondence.

To the Editor:

In following the discussion on the probable method of attachment of implanted teeth, I have noticed an uncertainty as to the possible result of the implantation of a tooth devoid of its pericemental membrane.

I have a case which will give positive data, and herewith submit it.

Patient, a lady of fine physique, aged say thirty, general health good, called March 22, 1887, suffering from a chronic abscess of the left superior second bicuspid root. Attempts at treatment and crowning having failed, and the outer plate of the alveolar wall being gone, and with fistulous opening, the remainder of the end of the root was extracted under gas on August 19, 1887.

The abscessed socket was treated with phenol sodique, wiped out with a pledget of cotton soaked with it, and left open to heal. On the 24th, five days later (I generally leave these cases ten days), the patient presented herself, and requested the implantation of a tooth, which in the mean time had been prepared in the following manner:

Not being able to find a bicuspid of proper color and form, a dry, healthy molar root was excised and a Logan crown fitted to it, and set in Weston's cement. This was put in the usual solution of bichloride of mercury (1 to 1000) and gradually heated up while distending the now contracted walls of the partially-healed socket, with cones of cotton. Imagine my surprise on examining my tooth to find the water almost boiling, and the pericementum entirely peeled off the distal surface of the root. The entire pericementum was now *dead* beyond possibility of resuscitation,—the cooked portion on one side remaining. The socket being prepared, I decided to insert the tooth to keep it open until another tooth could be prepared.

I have no memorandum of the date of the removal of the ligatures and plate which held the tooth; but on her return, several days later, the tooth was found to be attached so firmly that it was decided to leave it until it should become loose.

I have seen the tooth occasionally since, and to-day, after fifteen months of such service as an otherwise perfect set of teeth is subjected to, it has grown quite healthy and firm. By probing, an alveolar wall and healthy membranes were found entirely surrounding it at depths of two, three, and four millimetres above the gum margin, preventing the thin flat probe from entering farther, and giving healthy blood at each puncture. The side on which there was no pericementum, when the tooth was implanted, differed in no way from the other, and the patient uses this side of the arch almost exclusively, owing to the tenderness of the second bicuspid root on the opposite side, which was treated and crowned at the same time.

I have other cases under observation, one of an exactly similar character as regards the socket,—that of a seriously abscessed nature,—with absorbed outer alveolar wall, where a sound healthy tooth with good pericementum was implanted, ten days after the extraction of the diseased root. The development and restoration of the outer alveolar wall has been complete, and, after seventeen months' good service, has a firmness about it and a peculiar ring on percussion that would indicate the possibility of bony anchylosis.

The latter case was reported at the meeting of the American Dental Society of Europe, at Coblenz, August, 1887, it having been implanted March 15, of the same year, and being, to the best of my knowledge, the first case reported of the operation of implanting a *dry tooth* of indefinite antiquity in a natural socket. Dr. Elliott then volunteered the remark that the root would be absorbed, but judging from its appearance a month ago, when I filled a small cavity on its distal surface, caused by extensive decay on the mesial surface of the adjoining tooth, nature has not decided to take the course suggested by the president of the society.

In a correspondence with Professor Miller, he informs me that the best artificial medium for preserving pericementum or other animal tissue *alive*, is the physiological salt solution 0.75 table-salt to 100 water. He further says, "Under ordinary circumstances warm sublimate solution, 1 to 1000, will completely sterilize an old tooth in half an hour. If, however, the pericementum is thick and very dry and hard, I have my doubts about it.

When old teeth are used I see no reason at all why one should not use stronger solutions, say 5 to 1000; it cannot do any harm to the already dead pericementum.

To remove the sublimate before implanting, one can wash the tooth in water which has been sterilized by boiling.

Certain experiments, which may be easily repeated by any one, seem to prove that suitable disinfectants and antiseptics may with time be made to penetrate the entire substance of the dentine and cementum. Take a dry three-rooted molar, and into two of the roots loosely place cotton saturated with eugenol and seal it up; in from one to three days, as the dentine is porous or dense, the eugenol will be found to have penetrated the entire dentine and cementum of the roots which were treated, leaving them semi-transparent, while the root which had no dressing will remain white, dry, and opaque.

If we place a dry tooth in any *solution* of an antiseptic nature, weighing it before and after treatment, with a delicate scale, the amount absorbed will be found to vary with the density of the tooth-substance. By coloring the medicament with an aniline color and crushing the tooth afterwards the coloring-matter will be found to have penetrated the remotest lair of the dreaded bacteria. Twenty-four hours is the shortest time which a tooth should remain in an antiseptic fluid, owing to the slowness of the osmotic circulation through the dense membranes of the tooth-substance.

The most rapid absorption is from the pulp canal into the dentine. The cementum being less easily saturated. However, the fluids seem to penetrate wherever germ-life can exist. What we now most need is a suitable preparation for placing the teeth in immediately on extracting, that they may be kept in an antiseptic condition, and never allowed to become dry. When the tooth dries out, the dentine being more largely supplied with water, then the enamel seems to shrink more and injure the suture-like union between enamel and dentine, for even after back teeth have been resaturated the enamel readily chips away from the dentine and the whole crown is consequently weaker.

L. C. Bryan.

Basel, Switzerland.

